# Reproducibility of Preschool Personnel and Guardian Reports on Energy Balance-Related Behaviors and Their Correlates in Finnish Preschool Children

**DOI:** 10.3390/children5110144

**Published:** 2018-10-23

**Authors:** Suvi Määttä, Henna Vepsäläinen, Reetta Lehto, Maijaliisa Erkkola, Eva Roos, Carola Ray

**Affiliations:** 1 Samfundet Folkhälsan, Folkhälsan Research Center, 00250 Helsinki, Finland; suvi.maatta@folkhalsan.fi (S.M.); reetta.lehto@folkhalsan.fi (R.L.); Eva.roos@folkhalsan.fi (E.R.); 2 Department of Food and Nutrition, University of Helsinki, 00014 Helsinki, Finland; henna.vepsalainen@helsinki.fi (H.V.); maijaliisa.erkkola@helsinki.fi (M.E.); 3 Department of Public Health, Clinicum, University of Helsinki, 00014 Helsinki, Finland

**Keywords:** energy balance-related behaviors, preschool, children, reproducibility, test-retest reliability, diet, physical activity, screen time, food frequency questionnaire, correlates of energy balance-related behaviors

## Abstract

Valid and reliable non-objective assessments of guardian and preschool personnel reports are necessary when estimating young children’s health behaviors. This study examines the test-retest reproducibility of (a) guardian and preschool personnel questionnaires about correlates of preschool-aged children’s energy balance-related behaviors (EBRBs), (b) a screen time diary, and (c) a food frequency questionnaire (FFQ) reported by a guardian. A sample of guardians having preschool-aged children (*N* = 69) and preschool personnel (*N* = 61) completed questionnaires twice within a five-week time interval during April–May 2018 in Finland. Intra-class correlations (ICC), kappas, and percentage agreement were calculated to test the test-retest-reproducibility. The guardian questionnaire for correlates of the children’s EBRBs demonstrated mainly moderate to good reproducibility whereas the preschool personnel questionnaire of preschool correlates for children’s EBRBs was mostly good. The reproducibility of the screen time diary was good and FFQ food items showed mostly moderate reproducibility. The reproducibility of the FFQ foods items for vegetables, fruit, and berries was slightly better for the amount consumed than for the frequency of consumption. To conclude, all the instruments are acceptable for use in future studies.

## 1. Introduction

Early childhood is recognized as an important period in which to intervene and to prevent children becoming overweight or obese, or developing other risk factors for chronic diseases [1]. The reason for this importance is that children develop the habits of energy balance-related behaviors (EBRBs) that are based on the physical activity (PA), sedentary behavior (SB), and dietary behaviors at this age period [2,3]. According to recent reviews, regular PA at this age is associated with favorable motor development, fitness, and bone and skeletal health, improved cognitive development, and psychosocial and cardio metabolic health, whereas higher amounts of screen time, an indicator of SB, is associated primarily unfavorably with indicators of adiposity, motor or cognitive development, and psychosocial health [4,5]. In addition, a healthy diet is recommended to decrease the risk of non-communicable diseases and for instance, an excessive intake of sugary beverages has been show to influence BMI in children [6,7]. The EBRB habits learned at this age tend to track into later life [3,8]. A preschool-aged child, aged 3–6 years, learns most of these habits either at home or in preschool [9,10]. Home and preschool are, therefore, considered to be the two major settings for obesity prevention and health promotion in this age group [9,10]. Reliable and valid assessments of these settings are essential for estimating intervention effects. In addition, reliable assessments are needed in order to quantify the problems, monitor changes over time, and identify groups at high-risk.

In home and preschool settings, parents and preschool personnel play a significant role in the development of the children’s EBRB habits. For instance, they are essential role models about EBRBs for children, may limit or encourage for certain behavioral habits, and choose the availability of items for children (e.g., foods, screens, PA equipment) [11,12]. In addition, the developing cognitive capacity of children aged 3–6 questions the application of self-reporting measurements of EBRBs [13,14]. Consequently, it is necessary to approach parents or preschool personnel as surrogate reporters of children’s behaviors. In intervention studies, parents and preschool personnel often evaluate both the potential correlates of children’s EBRBs and the extent of children’s EBRBs. Thus, reproducibility of their reports may need to be estimated. The reproducibility of guardians’ questionnaires of correlates of children’s EBRBs has previously been reported in other studies [15,16,17], whereas reproducibility studies including both important children’s EBRBs settings, home, and preschool are, to our knowledge, needed.

As a part of the larger DAGIS project (Increased Health and Wellbeing in Preschools), a family-included preschool intervention promoting healthy EBRBs among children was conducted [18]. Since no established, reliable measures suitable for the Finnish context were available, the DAGIS study developed its own instruments to cover the relevant aspects of these settings. However, the reproducibility of these questionnaires was unknown.

This study examines the test-retest reproducibility of several separate questionnaires: (1) guardian and preschool personnel questionnaires on potential correlates of children’s EBRBs, (2) a children’s screen time diary reported by a guardian, and (3) a short food frequency questionnaire reported by a guardian.

## 2. Materials and Methods

The DAGIS research project has two major aims: (1) to recognize possible socioeconomic (SES) differences in children’s EBRBs and examine significant factors in the home and preschool setting associated with preschool children’s EBRBs and their possible SES differences, and (2) based on this knowledge, to develop and conduct a preschool intervention aiming to promote healthy EBRBs among all children, and to reduce potential SES differences in children’s EBRBs [18,19]. Throughout this project, socioecological models of health behaviors have guided the development of the questionnaires [20,21,22]. We assessed a range of parental and home factors with the guardian questionnaire, and similarly, a range of preschool factors with the preschool personnel questionnaire. Additionally, guardians filled in a screen time diary and a food frequency questionnaire (FFQ) measuring screen time and food consumption of their children.

As a part of the DAGIS project, a sub-study was conducted between February and May 2018. The aim of this sub-study was to examine the test-retest reproducibility of the questionnaires used in the DAGIS intervention study. The participants in this sub-study were recruited through preschools in one municipality in Southern Finland about 50 km from Helsinki. The participants had not been involved in the other parts of the DAGIS project. Of the eight preschools contacted, two were invited to participate only in the preschool personnel part, and six preschools were invited to participate in both preschool personnel and in the guardian part. Both preschools invited to participate only in the preschool part consented, and three preschools invited to participate both in preschool personnel and guardian part consented (63% of contacted preschools (5/8 preschools). A total of 118 out of 269 guardians consented on the behalf of their children to participate in this sub-study (consent rate 44%). Preschool personnel in the preschools were invited to the sub-study by receiving the questionnaires. It was possible for them to complete the paper-form questionnaire during working hours and 90 preschool personnel were invited. A total of 73 of them returned a completed questionnaire (81%).

The study procedures followed the following structure. All the research materials were delivered to the preschools by the DAGIS research staff, and preschool personnel at each preschool were asked to give them to the participating guardians. Guardians were asked to fill in three separate questionnaires during the measurement week: (1) a guardian questionnaire on potential home and parental correlates of children’s EBRBs, (2) a child’s 7-day screen time diary, and (3) a short FFQ on the child’s food intake. To lessen the burden on participants, the questionnaires were shortened versions of the ones used in the intervention study; including the most relevant questions in terms of the emphasis and needs of the DAGIS project. We provided an option to complete the guardian questionnaire and the FFQ in either paper or electronic form. Those who had chosen the electronic form got their questionnaires by an email. A small number of guardians had chosen to fill in the guardian’s questionnaire or the FFQ as a printed questionnaire. The screen time diary was in printed form. The preschool personnel filled in a questionnaire on potential correlates on children’s EBRBs at preschool in printed form. Both measurement weeks began on Monday and lasted for one week, both for guardians and for preschool personnel. There was a five-week time interval between the measurement weeks. Of the study material, it was pointed out multiple times that the same guardian should complete the questionnaires and diaries during both measurement weeks. The guardians who had not completed the questionnaires were at both assessment points reminded by emails to fill in one week after they had got the questionnaires. Both measurement weeks had a similar structure and conditions. The University of Helsinki Ethical Review Board in the Humanities and Social and Behavioral Sciences approved the study procedures (5/2018, approved in 16 March 2018).

### 2.1. The DAGIS Guardian Questionnaire

The questionnaire included items that were aimed to be influenced in the intervention and other important items from the intervention questionnaire. The development of the intervention questionnaire overall was a long-term process that included becoming familiar with previous research questions, having focus group interviews in 2014, pilot testing of questionnaires, and conducting a cross-sectional study in 2015–2016. More information of these phases can be read in the study protocol [18]. Based on the knowledge gained from these phases, the final form of the DAGIS intervention’s guardian questionnaire was created. Basically, the questionnaire assessed the potential home setting/parental correlates of the children’s EBRBs such as role modelling, availability and accessibility of foods and screens, rules and practices at home, attitudes, norms, parental self-efficacy, and children’s self-regulation skills. These factors have come up as potential influential factors of preschool children’s EBRBs in DAGIS focus group interviews [23,24,25]. They have also been recognized as influential factors for preschool children’s EBRBs in the DAGIS survey study [26], and in international studies [27,28,29]. To measure these factors in this questionnaire, we used questions that have been used in other studies [30,31,32,33], and in addition we developed our own items. Guardians also evaluated their child’s self-regulation skills on a 10-item instrument withholding two sub-categories which have been used earlier in another study [34]. Most items which were derived from international studies were translated and back translated during the cross-sectional study phase of the DAGIS study. The research group translated some items into Finnish particularly for the DAGIS intervention study.

Most of the concepts studied were assessed by one or two items and the concepts were assessed on a find-point scale with response categories ranging from e.g., ‘I fully agree’ to ‘I fully disagree’ or ‘always’ to ‘never’. The exact response scales of each item is reported in Supplement Table S1. In the analyses, we used most of the items as they were measured. However, some modifications were made in the answers on parental views about the suitable amount of PA, screen time, fruit, vegetables and sugary foods for 3–6-year old children. The suitable amount of PA and screen time, which had to be reported in hours and/or minutes, were transferred into minutes. Similarly, the suitable amount of the different food groups, which were asked to be reported either per week or per day, were transformed into per day. In the analyses, the items were treated as separate, but for result tables they were grouped into sections according to the behavior: sedentary behavior and screen time, physical activity, vegetables, fruit and berries, sugary foods and beverages, other food products, child’s self-regulation skills. The educational level of both guardians was assessed by a question about the highest educational achievement, with five response categories from lowest (comprehensive school) to highest (licentiate/doctorate) [19]. The guardian also reported the child’s age (years and months), their own age (years) and the child’s gender.

### 2.2. The DAGIS Preschool Personnel Questionnaire

The questionnaire included the key factors that the DAGIS intervention study focused on, such as self-efficacy for influencing children’s EBRBs, work-wellbeing, the self-regulation skills of children in the preschool group, frequency of discussion about the children’s EBRBs with guardians and knowledge of children’s health behavior recommendations. Work wellbeing items, except the evaluation of current stress level, were taken from QPSNordic [35], but we only took four sub-sections of this questionnaire and one additional item for our study. Most of the questions in this questionnaire were self-developed based on the results from a DAGIS focus group study [23,25]. Some questions were modified versions of previously used questions [36,37].

The factors studied were mostly assessed by using one or two items. Response categories for the items followed the same structure as the guardians’ questionnaire, a 5-point scale e.g., ranging from ‘I fully agree’ to ‘I fully disagree’ or ‘always’ to ‘never’. The exact response scales of each item is reported in Supplement Table S2. In the analyses, we used most of the items as they were measured. The items and answers regarding the suitable amount spent on children’s PA, screen time and intake of some foods were treated in the same manner as in the guardians’ questionnaire. The questionnaire also included a question about the highest formal pedagogical education of the responding preschool personnel.

### 2.3. The DAGIS Screen Time Diary

The screen time diary was a modified version of a diary, which has previously been validated against accelerometry [38]. However, to the best of our knowledge the reproducibility of this diary has not been tested before. We followed a structure similar to this validated diary, but we added portable screens into our diary and also measured more closely the habits related to screen use. Guardians were asked to complete their children’s screen time for seven days. On each day, guardians were asked to reply if their child did any of several activities today, how often and for how many hours and minutes. Guardians were asked to consider the following activities: TV viewing, computer use, DVD watching, tablet computer use and smart phone use. In addition, guardians were asked to report on the following screen time habits: if the child used screens one hour before bed time, and if the child had used screens with at least one of the guardians on that day. Similarly, guardians were asked to report the times and durations (in hours and minutes).

The guardian was asked to consider only out-of-preschool hours when reporting the child’s screen time. To be included in the analyses, we required guardians to fill in the diary for at least four days, and one of these days needed to be a weekend day. The total time reported for certain activities was transformed into minutes, and total minutes per day were calculated together. The weighted mean of weekday (5/7) and weekend (2/7) screen time in minutes was calculated to form the daily mean screen time. We also created the mean minutes of each screen type in similar manner. Similarly, we formed the minutes of screen use together with at least one guardian and screen use before bed time. In the analyses, we compared the mean minutes of total screen time, the mean minutes of separate screens, screen time before bedtime, and screen time together with at least one guardian between both measurement weeks.

### 2.4. The DAGIS Food Frequency Questionnaire (FFQ)

Since no culturally appropriate FFQs focusing on the food consumption of Finnish preschool-aged children existed, we developed a 47-item FFQ to measure food consumption among the preschoolers in the DAGIS survey [39,40]. The English version of the FFQ is available online [41]. The items included in the FFQ were based on earlier studies regarding the key contributors to the consumption of fruit and vegetables and the intake of added sucrose among Finnish children [42,43], but it also contained other foods or food groups to cover the whole diet of the children. In this sub-study, 25 items were used as these items were essential outcomes in the DAGIS intervention study. The 25-item FFQ concentrated on vegetables, fruit and berries as well as sugar-enriched foods. The guardians of the children reported how many times during the past week the child had consumed different foods at home or in places other than preschool. The FFQ excluded foods and beverages consumed during the preschool hours, since the parents would not have been able to assess these reliably. The FFQ included three answer columns: ‘not at all’, ‘times per week’ and ‘times per day’. The guardians were instructed to tick the ‘not at all’ column or to write a number in one of the other columns. For the analyses, food consumption data was converted into times per day.

In addition, the guardians reported the average daily consumption (in grams) of all but one food item (peas, beans, lentils and soya) included in the FFQ. This part of the FFQ was based on the tool developed in the ToyBox study [37,44] and modified to fit the emphasis and needs of the DAGIS study. To facilitate the estimation of the amounts, the guardians received a link to an electronic food picture booklet [45]. For the analyses, daily average amount (grams/day) was calculated based on both consumption frequency and the amount consumed.

### 2.5. The Statistical Methods

The agreement of the questionnaire items was analyzed with two-way mixed-effects single measurement intraclass correlation (ICC 3.1) model with absolute agreement, if the item could be treated as continuous. When selecting the ICC model, we followed the recommendations of Koo and Li [46]. With ordinal items we calculated weighted kappa [47]. In total 37 items in guardian questionnaire and 3 items in preschool personnel’s questionnaire were ordinal. To simplify the interpretation of the results, we used same criteria for ICCs and kappa values to classify the results. ICCs and kappa values were classified as excellent (≥0.81), good (0.61–0.80), moderate (0.41–0.60), and poor (≤0.40) [48,49,50]. The ICC values can be low when there is little variability between participants. To account for this potential misclassification of items as having low inter-rater reproducibility in the presence of little or no response variability, percentage agreement and weighted kappa was examined for items with poor ICC values. The criteria for percentage agreement are ‘excellent’ (90–100%), ‘good’ (75–89%), ‘moderate’ (60–74%), or ‘poor’ (<60%) [16,17].

Recently, it has also been recommended to pay attention to the confidence intervals (CI) of the ICC. Koo and Li [46] states that the level of reliability can be estimated by looking at the lower and upper limits of 95% confidence interval. In these estimations, we used the following criteria: 95% CI values less than 0.50 are poor, 95% CI values between 0.50 and 0.75 moderate reproducibility, 95% CI values between 0.75 and 0.90 good reproducibility, and 95% CI values greater than 0.90 indicate excellent reproducibility. This criteria means for instance that if the 95% confidence interval of an ICC is between 0.55 (lower 95% CI)–0.80 (upper 95% CI), the level of reliability can be regarded as ‘moderate’ to ‘good’ [46].

As items related to children’s self-regulation skills and preschool personnel’s’ work wellbeing measured by QPS Nordic are meant to be used as sum variables, we summed up the items as recommended in the guidelines [34,35]. All the statistical analyses were performed using Statistical Package for the Social Sciences (SPSS) (Version 25 SPSS Inc., Chicago, IL, USA).

## 3. Results

The characteristics of the guardians and preschool personnel who participated in the DAGIS test-retest reproducibility sub-study are shown in Table 1. In total, 99 (out of 118) guardian questionnaires were returned after the first measurement week. Of these, we did not receive 18 (18%) retest questionnaires five weeks later. In addition, one retest questionnaire was returned, but we had not received the guardian questionnaire from the first measurement week. We had 81 questionnaires with data from both measurement weeks (69% of the guardian questionnaires delivered in the first measurement week (81/118)).

We had screen time data from both measurement weeks for 72 children of 121 (59%). The screen time for one child was not reported during the weekend and was therefore excluded. We also included only the screen time data of one child in the case of siblings. Therefore, we had screen time data for 63 children to be used in the final analyses. We received food consumption data about 86 children in the first and about 67 children in the second measurement week. Altogether 62 children of 121, 51% of those who consented, provided FFQ data from both measurement weeks. Since the initial sample included multiple responses from the same guardians (sibling pairs), we only included data from 54 children in the final analyses. A total of 73 of the 90 invited preschool personnel responded to the questionnaire during the first measurement week (81%). Two members of the preschool personnel responded in the second measurement week but had not done so in the first measurement week. In addition, 12 people did not respond in the second measurement week. Therefore, a total of 61 preschool personnel responded to the questionnaire in both measurement weeks (response rate 68%).

### ICCs, Kappa Values, and Percentage Agreement

The ICCs of correlates and sociodemographic items are presented as sections (Table 2), each section including items related to a specific health behavior or socioeconomic status. The separate items of the guardian and the personnel questionnaires are presented in Supplementary Tables S1 and S2. Table 2 summarizes the observed ICCs per section in both measurements. Of the items in the guardian questionnaire, 42% (64/154) had moderate ICCs or kappa values, whereas 38% (59/154) had good ICCs or kappa values. Correlates of SB and screen time, PA and vegetable, berry, and fruit intake in the guardian questionnaire had mainly good ICC or kappa values whereas most of the items on correlates of sugar intake and self-regulation skills had moderate ICC or kappa values. All the sociodemographic items had an excellent test-retest reproducibility. Of the correlates of child’s EBRBs, the lowest ICC (0.086) was for the statement ‘I make sure that there are other activities available for my child to do instead of using electronic devices.’ The highest ICC (0.847) for the item ‘How often do you have the following foods at home? Fruit smoothies or purees with no added sugar’. When we summed up the self-regulation items into two sum variables, independence self-regulation sum variable had an ICC of 0.747 (0.619–0.836) whereas ICC of emotion dysregulation was 0.598 (0.420–0.731). When looking at the results of percentage agreement for the poor ICC values (*N* = 12), all the 12 items still had poor percentage agreement. The kappa values for these items with poor ICC values are reported in Supplement Table S1.

When we paid attention to the CIs of ICCs in the guardian questionnaire, CIs were between poor to moderate in 66% of the items (101/152). CIs were between moderate to good in 23% of the items (35/152). The CIs of the other items were ranked as poor (2%, 3/152), poor to good (6%, 9/152), moderate to excellent (1%, 1/152), and excellent (1%, 2/152) categories.

In the preschool personnel questionnaire, 44% of the items (19/43) had good ICC values. The section on correlates of vegetable, berry, and fruit intake had mainly poor ICC values. The section of correlates of SB and screen time was split between good and moderate. The section of correlates of sugar intake and PA was split between poor, moderate, and good. The section on parental views of child’s self-regulation skills had mainly moderate ICC values. All the sociodemographic items had excellent reproducibility. The section on the work wellbeing of preschool personnel had good reproducibility. When we summed up the items in work wellbeing section into four categories, the ICCs were as follows: support from co-workers 0.757 (0.625–0.847), support from superior 0.782 (0.661–0.863), innovative climate 0.702 (0.549–0.809), and perception of group work 0.816 (0.710–0.886). Of the items related to the children’s EBRBs, the highest value (0.877) was for the item ‘How often during the last week did you eat the same food at the same table with the children during lunch?’. The lowest value (0.156) was for the item ‘At the moment, to what extent can you impact the following things in your preschool? Children’s consumption of sugar-containing foods’. Since the calculation of ICCs depends on the existence of the variability in the answering categories, the ICCs for the following item in the preschool personnel questionnaire was not possible to calculate: ‘How often during the last week did you eat the same food as the children but not at the same table at lunch?’. However, we estimated its percentage agreement. When we paid attention to the poor ICC values (*N* = 9), one had excellent percentage agreement, one had good agreement, one moderate agreement and six had poor agreement. The kappa values for these items with poor ICC values are reported in Supplement Table S2.

When we paid attention into the CIs of ICCs in preschool personnel questionnaire, CIs were between poor to moderate in 37% of the items (16/43). CIs were between poor to good in 21% of the items (9/43), and between moderate to good in 21% of the items (9/43). The CIs of other items were ranked as poor (12%, 5/43), good to excellent (2%, 1/43), and excellent (7%, 3/43).

The ICC values for the screen time diary were at least good (50%, 4/8 items) (Supplement Table S3). The highest value (0.810) was for the average computer use whereas the lowest value (0.163) was for the item ‘average screen use before sleep’. The average total screen time had good ICC, and for the separate screen devices the ICCs were mainly good. The two items related to habits about screen time had either poor or moderate ICCs. The CIs of ICCs in the screen time diary were poor to moderate (50%, 4/8), moderate to good (38%, 3/8) and poor (12% 1/8).

Regarding the FFQ, the highest ICCs were observed for sugar, honey and/or syrup added to the food (Supplement Table S4; 0.758 when measured as times/day; 0.807 when measured as g/day), whereas reduced sugar juices and soft drinks had the lowest ICCs (0.000; −0.009). When food consumption was measured as times/day, 48% of items (12/25) were classified as having moderate reproducibility. No items were classified as having excellent reproducibility, whereas 20% of the items (5/25) had good reproducibility and 32% (8/25) poor reproducibility. Of the food items included in the vegetables, fruit, and berries, 38% (3/8) had good reproducibility, whereas 40% of items included in the dairy products (2/5), as well as in the cereal products (2/5) were classified as having poor reproducibility. Most of the CIs were classified as ‘poor to moderate’ (68%, 17/25), whereas 16% (4/25) of the CIs were ‘poor’, 8% (2/25) ‘moderate to good’, and 8% (2/25) ‘poor to good’.

When food consumption was measured as g/day, 54% of the items (13/24) had moderate reproducibility, whereas 25% (6/24) had good, 17% (4/24) poor, and 4% (1/24) excellent reproducibility. Vegetables, fruit, and berries were most reliably reported (43% of the items had good reproducibility). CIs were predominantly classified as ‘poor to moderate’ (63%, 15/24), whereas 17% (4/24) were ‘poor to good’, 13% (3/24) were ‘moderate to good’ and 8% (2/24) were ‘poor’. Higher ICCs were observed for calculated consumption than for consumption frequency of most vegetables, fruit and berries, cereal products, as well as for “others” (Supplement Table S4).

## 4. Discussion

The aim of this study was to examine the test-retest reproducibility of home and preschool correlates of children’s EBRBs, a screen time diary and a FFQ used in the DAGIS intervention study. To summarize our findings, a range of reproducibility evidence for the individual items and sections in the questionnaires existed in our instruments. The reproducibility of the guardian questionnaire on home correlates of children’s EBRBs was mainly moderate to good. The reproducibility of the preschool personnel questionnaire on preschool correlates of children’s EBRBs was mostly good. Similarly, the reproducibility of the screen time diary was good. FFQ food items showed mostly moderate reproducibility. The reproducibility of the FFQ foods items for vegetables, fruit, and berries was slightly better for the amount consumed than for the frequency of consumption.

The other reproducibility studies assessing parental correlates to children’s EBRBs have had stronger reproducibility results than the results of our study [15,16,17]. One reason for this may be that we had a time interval of five weeks in our study whereas the other studies had an interval of about two weeks between questionnaire measurements. However, there is no standard rule for the length of intervals [51]. Still, it is recommended not to administer a questionnaire after a very short interval as respondents may remember their previous responses [52]. Alternatively, when a longer interval is used, true changes in habits as well as variations in response might contribute to reduced reproducibility. In the review by Cade et al. [53], the time interval between repeat administrations of the FFQs ranged from two hours to 15 years and correlations were somewhat higher for questionnaires administered more proximal compared to those more distal. Memory may play a role in how well the questionnaire is answered, but interpretation or understanding of questions is also a factor affecting questionnaire reproducibility and the quality of the reports. For instance, some of the items with poor reproducibility in our study (e.g., we have sugary everyday foods at home, but they are not available for the child) may have been confusing to understand leaving an option for variation in answers between the measurement weeks. In addition, some of the items (e.g., we do not buy sugary treats to take home) may have produced better reproducibility with another response scale (currently from ‘totally disagree’ to ‘totally agree’ with ‘neither agree or disagree’ as the middle option instead of ‘yes’ or ‘no’). Previously, it has been reported that test-retest reproducibility seems to be lower when the distribution of responses on a scale is centered around a neutral-response midpoint indicating uncertainty in how to respond [54]. The ICC values of items may also have been higher if we examined them as summed totals. When we totaled two self-regulation variables and four work wellbeing categories according the guidelines [34,35], the reproducibility of these summed variables was higher than the reproducibility of the separate items.

Overall, about 25% of the items in the section of correlates of sugar intake had poor reproducibility, and 45% had moderate reproducibility. Besides the possible misunderstanding of these items or having an erroneous response scale, these items may also reflect the variable nature of sugar-related practices or the in-built difficulties in measuring them. On the other hand, items related to the correlates of SB, screen time, PA and vegetable, berry, and fruit intake had largely good reproducibility. Many of the items with the highest reproducibility were about the physical environment (e.g., availability of foods and PA equipment, PA places near home), which is usually quite stable over time. Therefore, these factors may be easier for guardians to report consistently over time.

A range of reproducibility evidence existed in the preschool personnel questionnaire. The work wellbeing section had good overall reproducibility whereas the sections related to correlates of sugar and vegetable, fruit, and berry intake had in many items poor reproducibility. The low reproducibility may not be due to the preschool personnel inconsistent reporting or misreporting rather it may reflect lack of true variation between preschools. In Finland, preschools are municipality-based, and the municipality can control mealtime practices and the content of meals served. The participating preschools in this study are situated in the same municipality and, therefore, these preschools may have similar practices related to EBRBs causing low between subject variations. The percentage agreement of these items with poor ICCs was better compared to ICC values supporting that there was no or little variation in answers between the preschool personnel. Therefore, it may be necessary to conduct a reproducibility study for the preschool personnel questionnaire among a sample including preschools from multiple municipalities.

Sociodemographic characteristics showed the overall highest reproducibility both in both the guardian and preschool personnel questionnaires. The information regarding sociodemographic characteristics is usually stable so only small differences can be expected to be found. In addition, the questions used in the DAGIS study are those commonly used in the Finnish context when determining sociodemographic characteristics, so it can be expected that the test-retest reproducibility should be good.

The reproducibility of the screen time diary was mainly good. Especially the total screen time and the average times of separate screens had good reproducibility. Only DVD use had a notably lower reproducibility compared to other screens. It may be that DVD use is not part of the habitual routines that happen similarly each week whereas there could be day-to-day-variability. For instance, if there is bad weather outside, children may be allowed more DVD use instead of other activities outside. DVD use may, therefore, replace other activities every now and then, whereas other screens have regular daily/weekly times when it is used. Similarly, other studies have also noted that some SBs may have irregular patterns and high between-week variability, although the total sedentary time may not vary from week to week [55].

The DAGIS FFQ showed mostly moderate reproducibility, and the ICCs varied between –0.009 and 0.807. A review by Kolodziejzcyk et al. stated that the mean correlation coefficients in test-retest studies have ranged from 0.40 to 0.83 [56]. However, most of the participants in the studies were adolescents. In addition, instead of ICCs or kappas, the studies reported mostly Spearman correlation coefficients, which, in fact, are not suitable for measuring the reproducibility of two measures. Other methodological aspects, such as answer options or categories may hinder the comparison of studies. Our slightly lower ICCs can be due to the fact that in order to best capture existing variations, we did not provide the participants with response categories. Consider the situation in which the child had eaten fruit four times a week during the first measurement week, and six times a week during the second week. Using an FFQ with response categories, these two measures could have been categorized into the same category, thus improving the reproducibility compared to our situation. In addition, different studies have grouped food items differently, further impeding the comparison between studies. Our approach of asking the respondent to provide average daily amounts of foods is cognitively complex and might be difficult for respondents not involved in cooking. In general, our results were in line with earlier studies showing predominantly moderate or good reproducibility among children [57,58,59,60,61,62,63].

In general, our study demonstrated acceptable reproducibility for fresh vegetables; cooked and/or canned vegetables; and fresh fruit, as well as most of the sugar-enriched foods and drinks. However, some foods, such as ice cream and reduced-sugar juices and/or soft drinks showed poor reproducibility. This is probably explained by seasonal changes in diet, since during the second measurement week, Finland was experiencing an unexpected heat wave, which might have affected both ice cream and drink consumption among the preschoolers. In addition, the time interval between the measurements in our study was relatively long: of the 14 studies conducted among children and adolescents reviewed in the review by Kolodziejzcyk et al., 11 had time intervals of three weeks or less [55]. Thus, our slightly lower ICCs might reflect actual changes in diet instead of being indicative of poor reproducibility.

In our study, we used two measurements for food consumption: times/day and grams/day. All in all, the two assessment methods showed relatively similar patterns of reproducibility: 68% of the items measured as times/day were classified as having moderate or good reproducibility, whereas the corresponding percentage was 79 when measured as g/day. However, for some items, estimating both the frequency and the amount eaten seemed to improve the reproducibility. This was the case for berries: the ICC was 0.252 (poor) when calculated based on the frequency only, whereas it was 0.692 (good) when calculated using g/day. It seems that the double-estimation (the guardians had to first estimate the frequency and then the average daily amount) makes the estimation more reliable. For some food items, it might be more practical to estimate the consumption in total daily amount instead of frequencies.

For reproducibility studies, it is recommended the inclusion of more than 50 participants [50]. Although we fulfilled this recommendation, our sample size was still quite small which also limits the possible analyses than can be conducted. We also acknowledge that our sample size is on the limits of adequate in the case of ICC [51]. We did not take into account the possibility of sibling participation during the recruitment phase. The collected data contained 16 sibling pairs, which might have decreased the variation in the data and as a consequence lowered the ICCs. Therefore, we decided to include only one sibling to the final analyses. We also acknowledge a limitation due to possible clustering in terms of reproducibility within preschools; personnel from one preschool being consistent in answers and another preschool not, which could have affected the ICCs and CIs. To improve the quality of our results, we could also have calculated Spearman-Brown’s prophecy formula to estimate that the reliability level of ≥0.80 would have been achieved. Although efforts were made to create strong possible instruments, we may still need to pay attention to these poor reproducibility items in future studies and when reporting the results of these items.

To our knowledge, this is one of the first times that the reproducibility of a preschool personnel questionnaire measuring correlates of children’s EBRBs has been assessed. Preschool personnel are those who spend their working hours regularly with children and, therefore, it is necessary to have reliable methods to measure their subjective viewpoint on the correlates of children’s EBRBs. Another strength was that in this study we estimated the test-retest reproducibility of several parental reports at the same time, and the guardian’s questionnaire, the screen time diary, and the FFQ contained both correlates of child’s EBRBs as well as behaviors themselves. This allowed us to compare these ICCs and it mimics the actual situation in the DAGIS intervention study when parents who participated in the study had to report several behaviors of their child and other measures and background information.

## 5. Conclusions

This study suggests that the test-retest reproducibility of the instruments used in the DAGIS study are acceptable. The instruments measured the preschool personnel’s and guardians’ correlates of preschool children’s EBRBs, preschool children’s screen time and intake of fruit, berries, vegetables, and sugary foods and beverages. Most of the items had at least moderate-to-good test-retest reproducibility (with a couple of exceptions). The test-retest reproducibility of children’s screen time was good and that of the FFQ was basically acceptable. However, the items with poor reproducibility rates may need to be treated cautiously in future studies. The results of this study should be taken into account when interpreting the findings of the DAGIS intervention study, as well as other studies using these examined items and instruments.

## Figures and Tables

**Table 1:** Descriptive statistics of participants in the DAGIS reproducibility sub-study in spring 2018.

Participant	Variable		Percentage (%)	Mean (SD)
Guardians (*N* = 69)				
	Gender: Female		86%	
	Age			36 (5.5)
	Education			
		Basic	3%	
		Vocational school	27%	
		High school	6%	
		Bachelor’s degree	38%	
		Master’s degree	26%	
	Child’s age			5.5 (1.2)
	Child’s gender: Girls		50%	
	Responded in paper form		23%	
Preschool personnel (*N* = 61)				
	Gender: Female		99%	
	Age			48 (9.5)
	Education			
		No formal pedagogical education	3%	
		Other pedagogical education	8%	
		Vocational school	56%	
		Bachelor’s degree or similar	33%	

**Table 2:** Overview of the intraclass correlation coefficients (ICC) and kappa values for education, occupation and gender for the sections in the guardian questionnaire, preschool personnel’s questionnaire, screen time diary, and food frequency questionnaire in the DAGIS reproducibility sub-study in spring 2018.

Measurement	Section	Number of Items	ICC/Kappa Minimum–Maximum	Excellent (≥0.81) *N* (%)	Good (0.61–0.80) *N* (%)	Moderate (0.40–0.60) *N* (%)	Poor (≤0.40) *N* (%)
Guardian’s questionnaire							
	Correlates of sedentary behavior and screen time	20	0.086–0.780	0 (0%)	11 (55%)	8(40%)	1 (5%)
	Correlates of physical activity *	23	0.284–0.760	0 (0%)	11 (48%)	8 (35%)	4 (17%)
	Correlates of vegetable, berry and fruit intake *	37	0.306–0.806	1 (3%)	18 (49%)	14 (38%)	4 (11%)
	Correlates of sugar intake *	47	0.146–0.796	0 (0%)	13 (28%)	22(47%)	12 (26%)
	Correlates of intake of other foods *	12	0.282–0.847	1(1%)	3 (25%)	6 (50%)	2 (2%)
	Child’s self-regulation skills	10	0.353–0.642	0 (0%)	3 (30%)	6 (60%)	1 (10%)
	Sociodemographic variables *	5	0.878–1.00	5 (100%)	0 (0%)	0 (0%)	0 (0%)
	**Overall**	**154**	**0.086–1.00**	**7 (4%)**	**59 (38%)**	**64 (42%)**	**24 (16%)**
Preschool personnel’s questionnaire							
	Correlates of sedentary behavior and screen time	4	0.443–0.695	0 (0%)	2 (50%)	2(50%)	0 (0%)
	Correlates of physical activity	3	0.255–0.624	0 (0%)	1 (33%)	1 (33%)	1 (33%)
	Correlates of vegetable, berry and fruit intake	5	0.199–0.619	0 (0%)	1 (20%)	1 (20%)	3 (60%)
	Correlates of sugar intake	6	0.156–0.772	0 (0%)	2 (33%)	2 (33%)	2 (33%)
	Eating habits	1	0.877	1 (100%)	0 (0%)	0 (0%)	0 (0%)
	Children’s self-regulation skills	7	0.311–0.678	0 (0%)	3 (43%)	3 (43%)	1 (14%)
	Work wellbeing	13	0.261–0.732	0 (0%)	10 (77%)	2 (15%)	1 (8%)
	Sociodemographic variables *	4	0.833–1.00	4 (100%)			
	**Overall**	**43**	**0.156–0.877**	**5 (12%)**	**19 (44%)**	**11 (26%)**	**8 (19%)**
Screen time diary							
	Total screen time	1	0.652		1 (100%)		
	Screen time of each device	5	0.311–0.810	1 (20%)	2 (40%)	1 (20%)	1 (20%)
	Habits related to screen time	2	0.163–0.373			1 (50%)	1 (50%)
	Overall	8	0.163–0.810	1 (13.5%)	3 (37.5%)	2(25%)	2 (25%)
Food consumption frequency (times/day)							
	Vegetables, fruit and berries	8	0.252–0.669	0 (0%)	3 (37.5%)	3 (37.5%)	2 (25%)
	Dairy products	5	0.149–0.719	0 (0%)	1 (20%)	2 (40%)	2 (40%)
	Cereal products	5	0.195–0.577	0 (0%)	0 (0%)	3 (60%)	2 (40%)
	Drinks	3	0.000–0.567	0 (0%)	0 (0%)	2 (66%)	1 (33%)
	Others	4	0.384–0.758	0 (0%)	1 (25%)	2 (50%)	1 (25%)
	**Overall**	**25**	**0.000–0.758**	**0 (0%)**	**5 (20%)**	**12 (48%)**	**8 (32%)**
Calculated food consumption (g/day)							
	Vegetables, fruit and berries	7	0.397–0.752	0 (0%)	3 (43%)	3 (43%)	1 (14%)
	Dairy products	5	0.255–0.626	0 (0%)	2 (40%)	2 (40%)	1 (20%)
	Cereal products	5	0.410–0.620	0 (0%)	1 (20%)	4 (80%)	0 (0%)
	Drinks	3	−0.009–0.579	0 (0%)	0 (0%)	2 (66%)	1 (33%)
	Others	4	0.349–0.807	1 (25%)	0 (0%)	2 (50%)	1 (25%)
	**Overall**	**24**	**−0.009–0.807**	**1** **(4%)**	**6 (25%)**	**13 (54%)**	**4 (17%)**

* This section includes kappa values.

## Supplementary Materials

The following are available online at http://www.mdpi.com/2227-9067/5/11/144/s1; Supplement Table S1. The intraclass correlations (ICCs) or kappa values (*κ*) of guardians’ questionnaire items in the DAGIS reproducibility sub-study; Supplement Table S2. The intraclass correlations (ICCs) or kappa values (*κ*) of preschool personnel’s questionnaire items in the DAGIS reproducibility sub-study; Supplement Table S3. The intraclass correlations (ICCs) of screen time diary in the DAGIS reproducibility sub-study; Supplement Table S4. The intraclass correlations (ICCs) of food consumption measures in the DAGIS reproducibility sub-study.
