# Supplementary material for: Reproducibility of Preschool Personnel and Guardian Reports on Energy Balance-Related Behaviors and Their Correlates in Finnish Preschool Children
Source: Children (Basel). 2018 Oct 23;5(11):144. doi: 10.3390/children5110144 (PMC6262525; doi:10.3390/children5110144)
Supplement: Supplementary file 1 [file children-05-00144-s001.pdf]

Supplement Table 1. The intraclass correlations (ICCs) or Kappa values (*κ)* of guardians' questionnaire items in the DAGIS reproducibility sub-study. If both values are reported, the order is ICC/ *κ*. If only Kappa value is presented, it is indicated in parenthesis after item.

Guardians' questionnaire		Response options	ICC/ κ	95 % confidence interval		*N*
**Sedentary behavior and screen time**						
1.	I am pleased with my child's screen time	1=strongly disagree; 2=somewhat disagree; 3=neither disagree or agree; 4=somewhat agree; 5=strongly agree	0.607	0.436	0.737	69
2.	How confident are you that you could do the following? I can say no to my child's requests to use screens even when the child has a tantrum.	1= not at all confident; 2= somewhat unconfident; 3= something between; 4= somewhat confident; 5= extremely confident	0.648	0.487	0.767	69
3.	Sometimes it seems like the only way to get my child to behave is to promise screen time as a reward.	1=strongly disagree; 2=somewhat disagree; 3=neither disagree or agree; 4=somewhat agree; 5=strongly agree	0.656	0.498	0.771	69
4.	It is important for me to limit my child's screen time.	1=strongly disagree; 2=somewhat disagree; 3=neither disagree or agree; 4=somewhat agree; 5=strongly agree	0.780	0.667	0.858	69
5.	It is important for me that screen time does not take time from child's physical activity.	1=strongly disagree; 2=somewhat disagree; 3=neither disagree or agree; 4=somewhat agree; 5=strongly agree	0.539	0.346	0.687	69
6.	It's important for my child to learn how to use electronic devices, because I'm not very good at using them myself.	1=strongly disagree; 2=somewhat disagree; 3=neither disagree or agree; 4=somewhat agree; 5=strongly agree	0.584	0.404	0.721	69
7.	I make sure that there are other activities available for my child to do instead of using electronic devices.	1=strongly disagree; 2=somewhat disagree; 3=neither disagree or agree; 4=somewhat agree; 5=strongly agree	0.086 / 0.031	-0.156/ -0.183	0.317 / 0.244	69
8.	There is pressure from society to purchase and use different electronic devices.	1=strongly disagree; 2=somewhat disagree; 3=neither disagree or agree; 4=somewhat agree; 5=strongly agree	0.619	0.450	0.746	69
9.	I think the suitable amount of screen time for 3-6 year olds on weekdays is at a maximum of X minutes	Open ended question	0.569	0.386	0.708	69

10.	I think the suitable amount of screen time for 3-6 year olds on weekends is at a maximum of X minutes	Open ended question	0.670	0.517	0.782	69
11.	At the moment, what kind of role model do you feel like you are to your child concerning healthy health behaviors? Screen use	1=not very good......5=very good	0.695	0.546	0.801	68
12.	In our family we have rules about when the child can use screens.	1=strongly disagree; 2=somewhat disagree; 3=neither disagree or agree; 4=somewhat agree; 5=strongly agree	0.579	0.397	0.716	69
13.	In our family we have rules about for how long the child can use screens.	1=strongly disagree; 2=somewhat disagree; 3=neither disagree or agree; 4=somewhat agree; 5=strongly agree	0.459	0.254	0.625	69
14.	The child is aware about the rules concerning screen time.	1=strongly disagree; 2=somewhat disagree; 3=neither disagree or agree; 4=somewhat agree; 5=strongly agree	0.635	0.464	0.759	69
15.	Using screens is allowed at table.	1=strongly disagree; 2=somewhat disagree; 3=neither disagree or agree; 4=somewhat agree; 5=strongly agree	0.712	0.574	0.811	69
16.	Portable screens (e.g. Tablets, phones) are kept in sight of the child.	1=strongly disagree; 2=somewhat disagree; 3=neither disagree or agree; 4=somewhat agree; 5=strongly agree	0.621	0.452	0.747	69
17.	About how many hours a day do YOU usually use electronic devices during leisure time when your child is around? During weekdays	1= not at all; 2= less than 30 min. 3= between 30 min and 1 hour; 4= 1-2 hours; 5= 3-4 hours; 6= 5 hours or more	0.724	0.577	0.823	69
18.	About how many hours a day do YOU usually use electronic devices during leisure time when your child is around? During weekends	1= not at all; 2= less than 30 min. 3= between 30 min and 1 hour; 4= 1-2 hours; 5= 3-4 hours; 6= 5 hours or more	0.549	0.363	0.694	69
19.	How often during the last 6 months have you discussed with the staff of your child's preschool your child's screen use?	1=not once; 2=1-2 times; 3=3-5 times; 4=monthly; 5=weekly	0.561	0.377	0.703	69

20.	How often during the last 6 months have you discussed children's screen use with other parents from your child's preschool?	1=not once; 2=1-2 times; 3=3-5 times; 4=monthly; 5=weekly	0.624	0.455	0.749	69
**Physical activity**						
1.	I am pleased with my child’s physical activity level	1=strongly disagree; 2=somewhat disagree; 3=neither disagree or agree; 4=somewhat agree; 5=strongly agree	0.609	0.435	0.738	69
2.	How confident are you that you could do the following? I can get my child to be active enough.	1= not at all confident; 2= somewhat unconfident; 3= something between; 4= somewhat confident; 5= extremely confident	0.486	0.285	0.646	69
3.	Sport as a hobby and the related costs (e.g. equipment, materials, subscription fees) are too expensive.	1=strongly disagree; 2=somewhat disagree; 3=neither disagree or agree; 4=somewhat agree; 5=strongly agree	0.755	0.626	0.842	69
4.	I think the suitable amount of daily physical activity for 3-6-year old children is at least X minutes	Open ended question	0.468	0.265	0.632	69
5.	At the moment, what kind of role model do you feel like you are to your child concerning healthy health behaviors? Physical activity	1=not very good……5=very good	0.760	0.640	0.844	69
6.	How often during the last month has your child visited the following places with at least one adult in the family? Nature/forest	1=not once; 2=1-3 times a month; 3=1-2 times a week; 4=3-6 times a week; 5=daily	0.634	0.467	0.758	67
7.	How often during the last month has your child visited the following places with at least one adult in the family? Park/playground	1=not once; 2=1-3 times a month; 3=1-2 times a week; 4=3-6 times a week; 5=daily	0.699	0.536	0.808	68
8.	How often during the last month has your child visited the following places with at least one adult in the family? Own yard	1=not once; 2=1-3 times a month; 3=1-2 times a week; 4=3-6 times a week; 5=daily	0.553	0.177	0.753	68

9.	How often during the last month has your child visited the following places with at least one adult in the family? Indoor exercise facility (e.g. Swimming hall, gym)	1=not once; 2=1-3 times a month; 3=1-2 times a week; 4=3-6 times a week; 5=daily	0.558	0.353	0.708	68
10.	Near our home there are places where I can make short excursions with my child.	1=strongly disagree; 2=somewhat disagree; 3=neither disagree or agree; 4=somewhat agree; 5=strongly agree	0.594	0.414	0.729	68
11.	There are outdoor areas, like parks, yards, and playgrounds, near my home where my child can play actively.	1=strongly disagree; 2=somewhat disagree; 3=neither disagree or agree; 4=somewhat agree; 5=strongly agree	0.759	0.636	0.844	68
12.	The outdoor areas in my neighborhood have plenty of swing sets, slides, or other play equipment my child can use.	1=strongly disagree; 2=somewhat disagree; 3=neither disagree or agree; 4=somewhat agree; 5=strongly agree	0.634	0.469	0.757	68
13.	The yard or area outside our home has plenty of room for my child to actively play games like tag or chase	1=strongly disagree; 2=somewhat disagree; 3=neither disagree or agree; 4=somewhat agree; 5=strongly agree	0.678	0.526	0.788	68
14.	My child has plenty of room to run around and burn off energy in our home.	1=strongly disagree; 2=somewhat disagree; 3=neither disagree or agree; 4=somewhat agree; 5=strongly agree	0.653	0.457	0.781	68
15.	How often during the last 6 months have you discussed with the staff of your child's preschool your child's physical activity?	1=not once; 2=1-2 times; 3=3-5 times; 4=monthly; 5=weekly	0.396 / 0.379	0.181 / 0.034	0.576 / 0.302	69
16.	How often during the last 6 months have you discussed children's physical activity with other parents from your child's preschool?	1=not once; 2=1-2 times; 3=3-5 times; 4=monthly; 5=weekly	0.716	0.577	0.814	69
17.	To what extent do you feel like the following things are a barrier to your child's physical activity/increasing physical activity? Lack of skills or means (Kappa value)	1=not a barrier; 2=is a barrier to some extent; 3=is a barrier to a great extent	0.698	0.479	0.918	67

18.	To what extent do you feel like the following things are a barrier to your child's physical activity/increasing physical activity? Lack of time (Kappa value)	1=not a barrier; 2=is a barrier to some extent; 3=is a barrier to a great extent	0.451	0.271	0.631	69
19.	To what extent do you feel like the following things are a barrier to your child's physical activity/increasing physical activity? Lack of support from the other parent. (Kappa value)	1=not a barrier; 2=is a barrier to some extent; 3=is a barrier to a great extent	0.492	0.305	0.679	66
20.	To what extent do you feel like the following things are a barrier to your child's physical activity/increasing physical activity? Lack of or distance to suitable places for physical activity (Kappa value)	1=not a barrier; 2=is a barrier to some extent; 3=is a barrier to a great extent	0.528	0.306	0.749	69
21.	To what extent do you feel like the following things are a barrier to your child's physical activity/increasing physical activity? Lack of PA equipment at home. (Kappa value)	1=not a barrier; 2=is a barrier to some extent; 3=is a barrier to a great extent	0.391	0.175	0.607	69
22.	To what extent do you feel like the following things are a barrier to your child's physical activity/increasing physical activity? Own coping. (Kappa value)	1=not a barrier; 2=is a barrier to some extent; 3=is a barrier to a great extent	0.390	0.217	0.563	69
23.	To what extent do you feel like the following things are a barrier to your child's physical activity/increasing physical activity? Child's resistance. (Kappa value)	1=not a barrier; 2=is a barrier to some extent; 3=is a barrier to a great extent	0.284	0.069	0.499	69
**Vegetables, berries and fruit**						
1.	I am pleased with my child’s vegetable consumption	1=strongly disagree; 2=somewhat disagree; 3=neither disagree or agree; 4=somewhat agree; 5=strongly agree	0.685	0.536	0.793	69

2.	I am pleased with my child's fruit and berry consumption	1=strongly disagree; 2=somewhat disagree; 3=neither disagree or agree; 4=somewhat agree; 5=strongly agree	0.675	0.522	0.786	69
3.	How confident are you that you could do the following? I can get my child to eat enough vegetables.	1= not at all confident; 2= somewhat unconfident; 3= something between; 4= somewhat confident; 5= extremely confident	0.709	0.568	0.809	69
4.	How confident are you that you could do the following? I can get my child to eat enough fruit and berries.	1= not at all confident; 2= somewhat unconfident; 3= something between; 4= somewhat confident; 5= extremely confident	0.792	0.684	0.866	68
5.	I show my child that I really like eating vegetables, fruit and berries.	1=strongly disagree; 2=somewhat disagree; 3=neither disagree or agree; 4=somewhat agree; 5=strongly agree	0.408	0.190	0.587	69
6.	My child does not like vegetables.	1=strongly disagree; 2=somewhat disagree; 3=neither disagree or agree; 4=somewhat agree; 5=strongly agree	0.773	0.656	0.853	69
7.	My child does not like fruit and berries.	1=strongly disagree; 2=somewhat disagree; 3=neither disagree or agree; 4=somewhat agree; 5=strongly agree	0.806	0.704	0.875	69
8.	It is important for me to make sure my child eats enough vegetables, fruit and berries.	1=strongly disagree; 2=somewhat disagree; 3=neither disagree or agree; 4=somewhat agree; 5=strongly agree	0.468	0.265	0.632	69
9.	I think the suitable amount of vegetables for 3-6-year old children is: portions a day	Open ended question	0.568	0.381	0.711	66
10.	I think the suitable amount of fruit and berries for 3-6-year old children is: portions a day	Open ended question	0.457	0.243	0.629	59
11.	How many different types of the following foods do you currently have at home? Fresh vegetables	Open ended question	0.733	0.601	0.826	68
12.	How many different types of the following foods do you currently have at home? Fresh fruit and/or berries	Open ended question	0.445	0.228	0.620	64
13.	How often do you have the following foods at home? Fresh vegetables	1= never; 2= rarely; 3= sometimes; 4= often; 5= always	0.692	0.500	0.811	69

14.	How often do you have the following foods at home? Frozen vegetables	1= never; 2= rarely; 3= sometimes; 4= often; 5= always	0.625	0.457	0.750	69
15.	How often do you have the following foods at home? Fresh fruit and/or berries	1= never; 2= rarely; 3= sometimes; 4= often; 5= always	0.504	0.304	0.661	69
16.	How often do you have the following foods at home? Frozen fruit and/or berries	1= never; 2= rarely; 3= sometimes; 4= often; 5= always	0.560	0.374	0.703	68
17.	If you have had fresh vegetables, have they been kept in sight of the child? (Kappa Value)	1=no; 2=yes	0.597	0.350	0.844	63
18.	If you have had fresh fruit, have they been kept in sight of the child? (Kappa Value)	1=no; 2=yes	0.306	0.065	0.547	66
19.	At the moment, what role model do you feel like you are to your child concerning healthy health behaviors? Consumption of fruit and vegetables	1=not very good......5=very good	0.705	0.561	0.807	68
20.	During the past week how often did you eat vegetables when you ate with your child?	1= never; 2= 1-2 times; 3= 3-4 times; 4= 5-6 times; 5= daily	0.642	0.478	0.762	69
21.	During the past week how often did you eat fruit or berries when you ate with your child?	1= never; 2= 1-2 times; 3= 3-4 times; 4= 5-6 times; 5= daily	0.501	0.303	0.657	69
22.	How often during the last 6 months have you discussed with the staff of your child's preschool your child's consumption of fruit and vegetables?	1=not once; 2=1-2 times; 3=3-5 times; 4=monthly; 5=weekly	0.464	0.256	0.631	69
23.	How often during the last 6 months have you discussed children's consumption of fruit and vegetables with other parents from your child's preschool?	1=not once; 2=1-2 times; 3=3-5 times; 4=monthly; 5=weekly	0.613	0.441	0.742	69
24.	To what extent do you feel like following things are a barrier to increasing your child's	1=not a barrier; 2=is a barrier to some extent; 3=is a barrier to a great extent	0.446	0.231	0.661	69

	consumption of vegetables? Lack of skills or means. (Kappa value)					
25.	To what extent do you feel like following things are a barrier to increasing your child's consumption of vegetables? The laboriousness of preparing vegetables. (Kappa value)	1=not a barrier; 2=is a barrier to some extent; 3=is a barrier to a great extent	0.370	0.156	0.585	69
26.	To what extent do you feel like following things are a barrier to increasing your child's consumption of vegetables? The expensiveness of vegetables (Kappa value)	1=not a barrier; 2=is a barrier to some extent; 3=is a barrier to a great extent	0.700	0.481	0.919	68
27.	To what extent do you feel like following things are a barrier to increasing your child's consumption of vegetables? Lack of support from the other parent. (Kappa value)	1=not a barrier; 2=is a barrier to some extent; 3=is a barrier to a great extent	0.546	0.345	0.748	67
28.	To what extent do you feel like following things are a barrier to increasing your child's consumption of vegetables? Low availability of vegetables at home. (Kappa value)	1=not a barrier; 2=is a barrier to some extent; 3=is a barrier to a great extent	0.361	0.146	0.576	69
29.	To what extent do you feel like following things are a barrier to increasing your child's consumption of vegetables? Child's resistance (Kappa value)	1=not a barrier; 2=is a barrier to some extent; 3=is a barrier to a great extent	0.373	0.201	0.546	69
30.	To what extent do you feel like following things are a barrier to increasing your child's consumption of vegetables? Parent(s) do not like vegetables. (Kappa value)	1=not a barrier; 2=is a barrier to some extent; 3=is a barrier to a great extent	0.653	0.433	0.872	69
31.	To what extent do you feel like following things are a barrier to increasing your child's	1=not a barrier; 2=is a barrier to some extent; 3=is a barrier to a great extent	0.633	0.430	0.836	69

	consumption of fruit? Lack of skills or means. (Kappa value)					
32.	To what extent do you feel like following things are a barrier to increasing your child's consumption of fruit? Laborousness of preparing fruit. (Kappa value)	1=not a barrier; 2=is a barrier to some extent; 3=is a barrier to a great extent	0.539	0.322	0.756	69
33.	To what extent do you feel like following things are a barrier to increasing your child's consumption of fruit? The expensiveness of fruit. (Kappa value)	1=not a barrier; 2=is a barrier to some extent; 3=is a barrier to a great extent	0.617	0.408	0.826	69
34.	To what extent do you feel like following things are a barrier to increasing your child's consumption of fruit? Lack of support from the other parent. (Kappa value)	1=not a barrier; 2=is a barrier to some extent; 3=is a barrier to a great extent	0.678	0.473	0.883	67
35.	To what extent do you feel like following things are a barrier to increasing your child's consumption of fruit? Low availability of fruit at home. (Kappa value)	1=not a barrier; 2=is a barrier to some extent; 3=is a barrier to a great extent	0.628	0.398	0.857	69
36.	To what extent do you feel like following things are a barrier to increasing your child's consumption of fruit? Child's resistance. (Kappa value)	1=not a barrier; 2=is a barrier to some extent; 3=is a barrier to a great extent	0.483	0.299	0.667	69
37.	To what extent do you feel like following things are a barrier to increasing your child's consumption of fruit? Parent(s) do not like fruit. (Kappa value)	1=not a barrier; 2=is a barrier to some extent; 3=is a barrier to a great extent	0.717	0.479	0.954	69
**Sugary foods and beverages**						
1.	I am pleased with my child’s consumption of sugary everyday foods	1=strongly disagree; 2=somewhat disagree; 3=neither disagree or agree; 4=somewhat agree; 5=strongly agree	0.518	0.320	0.672	69

2.	I am pleased with my child's consumption of sugary beverages	1=strongly disagree; 2=somewhat disagree; 3=neither disagree or agree; 4=somewhat agree; 5=strongly agree	0.616	0.445	0.744	68
3.	I am pleased with my child's consumption of sugary treats	1=strongly disagree; 2=somewhat disagree; 3=neither disagree or agree; 4=somewhat agree; 5=strongly agree	0.465	0.258	0.632	69
4.	How confident are you that you could do the following? I can say no to my child's requests for sugary everyday foods even when the child has a tantrum.	1= not at all confident; 2= somewhat unconfident; 3= something between; 4= somewhat confident; 5= extremely confident	0.525	0.331	0.676	69
5.	How confident are you that you could do the following? I can say no to my child's requests for sugary beverages even when the child has a tantrum.	1= not at all confident; 2= somewhat unconfident; 3= something between; 4= somewhat confident; 5= extremely confident	0.493	0.290	0.653	69
6.	How confident are you that you could do the following? I can say no to my child's requests for sugary treats even when the child has a tantrum.	1= not at all confident; 2= somewhat unconfident; 3= something between; 4= somewhat confident; 5= extremely confident	0.504	0.305	0.661	69
7.	Sometimes it seems like the only way to get my child to behave is to promise sugary foods, beverages or treats as treats.	1=strongly disagree; 2=somewhat disagree; 3=neither disagree or agree; 4=somewhat agree; 5=strongly agree	0.751	0.627	0.838	69
8.	It is important for me to limit my child's consumption of sugary everyday foods.	1=strongly disagree; 2=somewhat disagree; 3=neither disagree or agree; 4=somewhat agree; 5=strongly agree	0.689	0.541	0.795	69
9.	It is important for me to limit my child's consumption of sugary beverages.	1=strongly disagree; 2=somewhat disagree; 3=neither disagree or agree; 4=somewhat agree; 5=strongly agree	0.146 / 0.292	-0.086 / 0.075	0.366 / 0.509	69
10.	It is important for me to limit my child's consumption of sugary treats.	1=strongly disagree; 2=somewhat disagree; 3=neither disagree or agree; 4=somewhat agree; 5=strongly agree	0.470	0.266	0.635	68
11.	I think the suitable amount of sugary everyday foods for 3-6-year old children is:	Open ended question	0.377 / 0.219	0.153 / 0.110	0.564 / 0.328	68

12.	I think the suitable amount of sugary drinks for 3-6-year old children is:	Open ended question	0.426	0.193	0.613	59
13.	I think the suitable amount of sugary treats for 3-6-year old children is:	Open ended question	0.388 / 0.310	0.172 / 0.191	0.570 / 0.429	68
14.	How often do you have the following foods at home? Yoghurt, quark etc. (also vegetable-based with added sugar	1= never; 2= rarely; 3= sometimes; 4= often; 5= always	0.625	0.457	0.749	69
15.	How often do you have the following foods at home? Pudding	1= never; 2= rarely; 3= sometimes; 4= often; 5= always	0.748	0.620	0.838	69
16.	How often do you have the following foods at home? Sugar-sweetened cereals or muesli (more than 10g/100g sugar)	1= never; 2= rarely; 3= sometimes; 4= often; 5= always	0.712	0.573	0.811	69
17.	How often do you have the following foods at home? Syrup with added sugar	1= never; 2= rarely; 3= sometimes; 4= often; 5= always	0.631	0.464	0.755	69
18.	How often do you have the following foods at home? Soft drinks with added sugar	1= never; 2= rarely; 3= sometimes; 4= often; 5= always	0.593	0.415	0.727	69
19.	How often do you have the following foods at home? Berry sauce with added sugar	1= never; 2= rarely; 3= sometimes; 4= often; 5= always	0.796	0.688	0.869	67
20.	How often do you have the following foods at home? Cakes, muffins, buns and sweet pastry	1= never; 2= rarely; 3= sometimes; 4= often; 5= always	0.389 / 0.380	0.171 / 0.222	0.571/ 0.539	69
21.	How often do you have the following foods at home? Cookies	1= never; 2= rarely; 3= sometimes; 4= often; 5= always	0.722	0.585	0.819	
22.	How often do you have the following foods at home? Chocolate and sweets	1= never; 2= rarely; 3= sometimes; 4= often; 5= always	0.355 / 0.254	0.128 / 0.109	0.546 / 0.398	68
23.	How often do you have the following foods at home? Ice cream	1= never; 2= rarely; 3= sometimes; 4= often; 5= always	0.370 / 0.185	0.134 / 0.050	0.564 / 0.320	69

24.	How often do you have the following foods at home? Jam	1= never; 2= rarely; 3= sometimes; 4= often; 5= always	0.629	0.461	0.753	69
25.	If you have had Sugar-sweetened cereals or muesli (more than 10g/100g sugar), have they been kept in sight of the child? (Kappa value)	1=no; 2=yes	0.661	0.406	0.916	59
26.	If you have had syrups with added sugar, has it been kept in sight of the child? (Kappa value)	1=no; 2=yes	0.486	0.241	0.731	64
27.	If you have had soft drinks with added sugar, have they been kept in sight of the child? (Kappa value)	1=no; 2=yes	0.576	0.327	0.825	62
28.	If you have had cookies etc., have they been kept in sight of the child? (Kappa value)	1=no; 2=yes	0.376	0.120	0.631	52
29.	If you have had sweet pastries, have they been kept in sight of the child? (Kappa value)	1=no; 2=yes	0.308	0.059	0.557	59
30.	If you have had chocolate and sweets, have they been kept in sight of the child? (Kappa value)	1=no; 2=yes	0.433	0.188	0.678	62
31.	At the moment, what kind of role model do you feel like you are to your child concerning healthy health behaviors? Consumption of sugary foods	1=not very good......5=very good	0.540	0.346	0.689	68
32.	At the moment, what kind of role model do you feel like you are to your child concerning healthy health behaviors? Consumption of sugary beverages	1=not very good......5=very good	0.439	0.226	0.613	68
33.	At the moment, what kind of role model do you feel like you are to your child concerning healthy health behaviors? Consumption of sugary treats	1=not very good......5=very good	0.418	0.199	0.597	68

34.	During the past week how often did you eat sugary everyday foods, when you ate with your child?	1= never; 2= 1-2 times; 3= 3-4 times; 4= 5-6 times; 5= daily	0.531	0.337	0.681	69
35.	During the past week how often did you eat sugary treats, when you ate with your child?	1= never; 2= 1-2 times; 3= 3-4 times; 4= 5-6 times; 5= daily	0.383 / 0.231	0.166 / 0.076	0.566 / 0.386	69
36.	During the past week how often did you drink sugary beverages, when you ate with your child?	1= never; 2= 1-2 times; 3= 3-4 times; 4= 5-6 times; 5= daily	0.441	0.232	0.612	69
37.	We do not buy sugary everyday foods at home.	1=strongly disagree; 2=somewhat disagree; 3=neither disagree or agree; 4=somewhat agree; 5=strongly agree	0.520	0.321	0.674	68
38.	We have sugary everyday foods at home, but they are not available to the child.	1=strongly disagree; 2=somewhat disagree; 3=neither disagree or agree; 4=somewhat agree; 5=strongly agree	0.286 / 0.160	0.054 / 0.034	0.488 / 0.285	69
39.	We allow only a certain amount of sugary everyday foods per day/week to the child.	1=strongly disagree; 2=somewhat disagree; 3=neither disagree or agree; 4=somewhat agree; 5=strongly agree	0.292 / 0.139	0.060 / -0.011	0.494 / 0.290	69
40.	We do not buy sugary beverages at home.	1=strongly disagree; 2=somewhat disagree; 3=neither disagree or agree; 4=somewhat agree; 5=strongly agree	0.706	0.564	0.807	69
41.	We have sugary beverages at home, but they are not available to the child.	1=strongly disagree; 2=somewhat disagree; 3=neither disagree or agree; 4=somewhat agree; 5=strongly agree	0.458	0.249	0.626	69
42.	We allow only a certain amount of sugary beverages per day/week to the child.	1=strongly disagree; 2=somewhat disagree; 3=neither disagree or agree; 4=somewhat agree; 5=strongly agree	0.669	0.516	0.781	69
43.	We do not buy sugary treats at home.	1=strongly disagree; 2=somewhat disagree; 3=neither disagree or agree; 4=somewhat agree; 5=strongly agree	0.237 / 0.286	-0.001 / 0.148	0.448 / 0.424	69
44.	We have sugary treats at home, but they are not available to the child.	1=strongly disagree; 2=somewhat disagree; 3=neither disagree or agree; 4=somewhat agree; 5=strongly agree	0.566	0.382	0.707	69
45.	We allow only a certain amount of sugary treats per day/week to the child.	1=strongly disagree; 2=somewhat disagree; 3=neither disagree or agree; 4=somewhat agree; 5=strongly agree	0.478	0.273	0.642	69

46.	How often during the last 6 months have you discussed with the staff of your child's preschool your child's consumption of sugary foods and beverages?	1=not once; 2=1-2 times; 3=3-5 times; 4=monthly; 5=weekly	0.477	0.274	0.640	69
47.	How often during the last 6 months have you discussed children's consumption of sugary foods and beverages with other parents from your child's preschool?	1=not once; 2=1-2 times; 3=3-5 times; 4=monthly; 5=weekly	0.601	0.426	0.733	69
**Child's self-regulation skills**						
1.	My child Does not need much help with tasks.	1=not true; 2=somewhat true; 3=certainly true;	0.458	0.252	0.625	69
2.	My child Persists in the face of difficult tasks	1=not true; 2=somewhat true; 3=certainly true;	0.639	0.473	0.761	68
3.	My child Likes to work things out for her/himself; seeks help only when necessary, or as a last resort	1=not true; 2=somewhat true; 3=certainly true;	0.415	0.201	0.592	69
4.	My child Can move to a new activity after finishing a task	1=not true; 2=somewhat true; 3=certainly true;	0.510	0.304	0.668	69
5.	My child Chooses activities on their own	1=not true; 2=somewhat true; 3=certainly true;	0.626	0.459	0.750	69
6.	My child Shows wide mood swings	1=not true; 2=somewhat true; 3=certainly true;	0.594	0.417	0.727	69
7.	My child Is easily frustrated	1=not true; 2=somewhat true; 3=certainly true;	0.548	0.359	0.694	69
8.	My child Is impulsive, acts without thinking	1=not true; 2=somewhat true; 3=certainly true;	0.642	0.477	0.763	68
9.	My child Gets over excited	1=not true; 2=somewhat true; 3=certainly true;	0.427	0.212	0.602	69
10.	My child Gets over being upset quickly	1=not true; 2=somewhat true; 3=certainly true;	0.353	0.128	0.543	69
**Other food products**						

1.	How often do you have the following foods at home? Yoghurt, quark etc. (also vegetable-based) without added sugar	1= never; 2= rarely; 3= sometimes; 4= often; 5= always	0.634	0.468	0.757	69
2.	How often do you have the following foods at home? Unseasoned nuts, almonds and/or seeds.	1= never; 2= rarely; 3= sometimes; 4= often; 5= always	0.767	0.650	0.849	69
3.	How often do you have the following foods at home? Unseasoned oatmeal etc.	1= never; 2= rarely; 3= sometimes; 4= often; 5= always	0.476	0.274	0.638	69
4.	How often do you have the following foods at home? Cereals with no or little added sugar (less than 10g/100g sugar)	1= never; 2= rarely; 3= sometimes; 4= often; 5= always	0.426	0.212	0.601	69
5.	How often do you have the following foods at home? Fruit smoothies or purees with no added sugar	1= never; 2= rarely; 3= sometimes; 4= often; 5= always	0.847	0.763	0.903	67
6.	How often do you have the following foods at home? Juice with no or little added sugar	1= never; 2= rarely; 3= sometimes; 4= often; 5= always	0.563	0.372	0.707	68
7.	How often do you have the following foods at home? Soft drinks with no or little added sugar	1= never; 2= rarely; 3= sometimes; 4= often; 5= always	0.692	0.545	0.798	69
8.	If you have had unseasoned nuts, almonds or seeds, have they been kept in sight of the child? (Kappa value)	1=no; 2=yes	0.451	0.203	0.700	62
9.	If you have had unseasoned oatmeal, has it been kept in sight of the child? (Kappa value)	1=no; 2=yes	0.500	0.255	0.745	64
10.	If you have had cereals with no or little added sugar (less than 10g/100g sugar), have they been kept in sight of the child? (Kappa value)	1=no; 2=yes	0.311	0.049	0.573	56

11.	If you have had juice with no or little added sugar, have they been kept in sight of the child? (Kappa value)	1=no; 2=yes	0.513	0.246	0.779	54
12.	If you have had soft drinks with no or little added sugar, have they been kept in sight of the child? (Kappa value)	1=no; 2=yes	0.283	0.011	0.555	69
**Sociodemographic**						
1.	Your age	Open ended question	0.997	0.995	0.998	68
2.	Child's age	Open ended question	1.00	1.00	1.00	69
3.	Child's gender (Kappa value)	1=girl, 2=boy	1.00	0.764	1.236	69
4.	Your educational level (Kappa value)	1=Comprehensive school, 2=Vocational school, 3=High school, 4=Bachelor's degree / college, 5=Master's degree, 6=Licentiate/doctor, 7=else, what	0.900	0.760	1.040	69
5.	The educational level of the spouse (if having) (Kappa value)	1=Comprehensive school, 2=Vocational school, 3=High school, 4=Bachelor's degree / college, 5=Master's degree, 6=Licentiate/doctor, 7=else, what	0.878	0.729	1.027	56
**Other**						
1.	How stressed out do you feel at the moment on a scale between 1-10?	1=not at all stressed out, 10= very stressed out	0.439	0.228	0.612	67

Supplement Table 2. The intraclass correlations (ICCs) or Kappa values (*κ*) of preschool personnel questionnaire items in the DAGIS reproducibility sub-study. If both values are reported, the order is ICC/ *κ*. If only Kappa value is presented, it is indicated in parenthesis after item.

**Preschool personnels' questionnaire**	Response options	ICC / *κ*	95 % confidence interval		*N*
**Sedentary behavior and screen time**					
1. At the moment, to what extent can you impact the following things in your preschool? Children's screen use	1=not at all; 2= not much; 3=to some extent; 4=quite a lot; 5=very much	0.676	0.509	0.794	59
2. How often during the last 6 months have you discussed with the parents of the children in your group about the child's screen use?	1=not once; 2=1-2 times; 3=3-5 times; 4=monthly; 5=weekly	0.443	0.213	0.627	59
3. I think the suitable amount of screen time on weekdays for 3-6-year-olds is at a maximum of: X minutes per day	Open ended question	0.587	0.390	0.732	59
4. I think the suitable amount of screen time on weekends for 3-6-year-olds is at a maximum of: X minutes per day	Open ended question	0.695	0.538	0.805	60
**Physical activity**					
1. At the moment, to what extent can you impact the following things in your preschool? Children's physical activity	1=not at all; 2= not much; 3=to some extent; 4=quite a lot; 5=very much	0.624	0.436	0.759	59
2. How often during the last 6 months have you discussed with the parents of the children in your group about the child's physical activity?	1=not once; 2=1-2 times; 3=3-5 times; 4=monthly; 5=weekly	0.496	0.275	0.666	59
3. I think that according to recommendations 3-6-year-old children should be physically active at least X minutes per day	Open ended question	0.255 / 0.211	0.004 / 0.069	0.476 / 0.353	60
**Vegetables, berries and fruit**					
1. At the moment, to what extent can you impact the following things in your preschool? Children's vegetable consumption	1=not at all; 2= not much; 3=to some extent; 4=quite a lot; 5=very much	0.199 / 0.171	-0.060 / -0.012	0.431 / 0.355	60

2.	At the moment, to what extent can you impact the following things in your preschool? Children's fruit and berry consumption	1=not at all; 2= not much; 3=to some extent; 4=quite a lot; 5=very much	0.346 / 0.420	0.102 / 0.237	0.551 / 0.603	59
3.	How often during the last week did you encourage the children to eat a variety of vegetables, fruit and berries in a versatile manner?	1=not once; 2=once; 3=twice; 4=3-4 times; 5=every day	0.256 / 0.225	0.014 / 0.060	0.474 / 0.391	59
4.	How often during the last 6 months have you discussed with the parents of the children in your group about the child's consumption of vegetables, fruit and berries?	1=not once; 2=1-2 times; 3=3-5 times; 4=monthly; 5=weekly	0.583	0.385	0.730	59
5.	I think that according to nutrition recommendations 3-6-year-old children should eat vegetables, fruit and berries daily, a minimum of: X portions per day.	Open ended question	0.619	0.432	0.755	59
**Sugary foods and beverages**						
1.	At the moment, to what extent can you impact the following things in your preschool? Children's consumption of sugar-containing foods	1=not at all; 2= not much; 3=to some extent; 4=quite a lot; 5=very much	0.156 / 0.064	-0.100 / -0.087	0.393 / 0.215	60
2.	At the moment, to what extent can you impact the following things in your preschool? Children's consumption of sugar-sweetened beverages	1=not at all; 2= not much; 3=to some extent; 4=quite a lot; 5=very much	0.206 / 0.131	-0.052 / -0.004	0.437 / 0.266	59
3.	How often during the last 6 months have you discussed with the parents of the children in your group about the child's consumption of sugar-containing food and drinks?	1=not once; 2=1-2 times; 3=3-5 times; 4=monthly; 5=weekly	0.422	0.194	0.609	58
4.	I think the suitable amount of sugary everyday food for 3-6-year-olds is at a maximum of:	Open ended question	0.605	0.411	0.747	57
5.	I think the suitable amount of sugary treats for 3-6-year-olds is at a maximum of:	Open ended question	0.537	0.324	0.699	57

6. I think the suitable amount of sugary drinks for 3-6-year-olds is at a maximum of:	Open ended question	0.772	0.634	0.862	52
**Eating**					
1. How often during the last week did you eat the same food at the same table with the children during lunch?		0.877	0.802	0.925	59
2. How often during the last week did you eat the same food as the children but not in the same table at lunch? (Kappa value)		-0.009	-0.268	0.251	59
**Self-regulation skills**					
1. I have enough means and capability to support children's self-regulation skills.	1=strongly disagree; 2=somewhat disagree; 3=neither disagree or agree; 4=somewhat agree; 5=strongly agree	0.631	0.451	0.762	59
2. I can support my group's children to calm down and concentrate.	1=strongly disagree; 2=somewhat disagree; 3=neither disagree or agree; 4=somewhat agree; 5=strongly agree	0.678	0.513	0.795	60
3. I can support my group's children to recognize their emotions.	1=strongly disagree; 2=somewhat disagree; 3=neither disagree or agree; 4=somewhat agree; 5=strongly agree	0.311 / 0.359	0.061 / 0.151	0.524 / 0.568	60
4. In my group, difficulties in emotion regulation are very common.	1=strongly disagree; 2=somewhat disagree; 3=neither disagree or agree; 4=somewhat agree; 5=strongly agree	0.486	0.266	0.660	58

5. In my group it is very common that calming down and concentrating is difficult.	1=strongly disagree; 2=somewhat disagree; 3=neither disagree or agree; 4=somewhat agree; 5=strongly agree	0.433	0.197	0.621	58
6. Overall, I am satisfied with the self-regulation skills of the children in my group.	1=strongly disagree; 2=somewhat disagree; 3=neither disagree or agree; 4=somewhat agree; 5=strongly agree	0.615	0.428	0.751	60
7. How often during the last 6 months have you discussed with the parents of the children in your group about the child's self-regulation?	1=not once; 2=1-2 times; 3=3-5 times; 4=monthly; 5=weekly	0.541	0.333	0.699	59
**Work wellbeing**					
1. On a scale of 1-10, how stressed out are you about your work?	1=not at all stressed out, 10= very stressed out	0.577	0.356	0.738	48
2. If needed, can you get support and help with your work from your co-workers?	1=Rarely or never; 2= quite rarely; 3= sometimes; 4=quite often; 5=very often or always;	0.650	0.478	0.775	60
3. If needed, can you get support and help with your work from your immediate superior?	1=Rarely or never; 2= quite rarely; 3= sometimes; 4=quite often; 5=very often or always;	0.723	0.577	0.824	61
4. If needed, is your immediate willing to listen to your task-related problems?	1=Rarely or never; 2= quite rarely; 3= sometimes; 4=quite often; 5=very often or always;	0.616	0.432	0.751	61
5. If needed, are your co-workers willing to listen to your work-related problems?	1=Rarely or never; 2= quite rarely; 3= sometimes; 4=quite often; 5=very often or always;	0.693	0.536	0.804	61

6. Are your work achievements appreciated by your immediate superior?	1=Rarely or never; 2= quite rarely; 3= sometimes; 4=quite often; 5=very often or always;	0.677	0.515	0.793	61
7. Do the workers take initiatives at your preschool?	1=Rarely or never; 2= quite rarely; 3= sometimes; 4=quite often; 5=very often or always;	0.698	0.544	0.807	61
8. Are workers encouraged to think of ways to do things better at your workplace?	1=Rarely or never; 2= quite rarely; 3= sometimes; 4=quite often; 5=very often or always;	0.261 / 0.189	0.010 / -0.001	0.480 / 0.379	61
9. Do you appreciate your membership of your group?	1=Rarely or never; 2= quite rarely; 3= sometimes; 4=quite often; 5=very often or always;	0.411	0.183	0.599	60
10. Is the management of your organization interested in the health and well-being of the personnel?	1=Rarely or never; 2= quite rarely; 3= sometimes; 4=quite often; 5=very often or always;	0.677	0.505	0.796	60
11. Is there sufficient communication within your work community?	1=Rarely or never; 2= quite rarely; 3= sometimes; 4=quite often; 5=very often or always;	0.661	0.492	0.782	61
12. Does your group or team work flexibly?	1=Rarely or never; 2= quite rarely; 3= sometimes; 4=quite often; 5=very often or always;	0.730	0.586	0.829	60
13. Is your group or team successful at problem solving?	1=Rarely or never; 2= quite rarely; 3= sometimes; 4=quite often; 5=very often or always;	0.732	0.589	0.831	60
**Sociodemographic**					
1. Your education concerning early childhood education (Kappa value)	1=Master's Degree (in education, specialized in ECE); 2=Bachelor's in education (Kindergarten teacher, University); 3=bachelor of social services; 4=Kindergarten	0.865	0.740	0.989	60

		teacher, college-level training; 5=Social pedagogue/educator (college-level); 6=Vocational qualification in social and health care (nursery nurse); 7= Else, what				
2.	What is your occupation? (Kappa value)	1=early childhood special teacher; 2= kindergarten teacher; 3=nursery nurse; 4=assistant; 5=Else, what;	0.833	0.671	0.994	60
3.	Gender (Kappa value)	1=woman; 2=man; 3=I don't want to answer	1.00	0.749	1.251	61
4.	Age	Open ended question	0.994	0.990	0.996	60

Supplement Table 3. The intraclass correlations (ICCs) of screen time diary in the DAGIS reproducibility sub-study.

**Screen time diary***	ICC	95% confidence interval		*N*
Average screen time	0.652	0.480	0.776	61
Average TV use	0.726	0.588	0.823	65
Average DVD use	0.311	0.072	0.515	65
Average tablet use	0.769	0.648	0.853	65
Average smart phone use	0.575	0.385	0.717	65
Average computer use	0.810	0.707	0.880	65
Average screen use together with at least one guardian	0.373	0.148	0.562	65
Average screen use before sleep	0.163	-0.086	0.391	65

*The guardians were asked to report how many hours and minutes child had done the listed activities per day.

Supplement Table 4. The intraclass correlation coefficients (ICCs) of food consumption measures in the DAGIS reproducibility sub-study.

	Food consumption frequency * (times/day)				Calculated food consumption (g/day)			
	ICC	95% confidence interval		*N*	ICC	95% confidence interval		*N*
**Vegetables, fruit and berries**								
Fresh vegetables	0.472	0.236	0.655	54	0.671	0.493	0.795	54
Cooked and canned vegetables	0.607	0.407	0.751	54	0.424	0.178	0.620	54
Peas, beans, lentils and soya	0.402	0.153	0.603	54	-	-	-	-
Fresh fruit	0.669	0.485	0.797	51	0.752	0.603	0.850	51
Canned and frozen fruit	0.479	0.232	0.669	49	0.509	0.282	0.682	54
Berries	0.252	-0.022	0.492	51	0.692	0.518	0.811	52
Dried fruit and berries	0.273	-0.010	0.515	48	0.397	0.142	0.603	52
Commercial baby foods and smoothies	0.644	0.437	0.786	46	0.549	0.327	0.714	52
**Dairy products**								
Flavored and sweetened milk- and plant-based drinks	0.572	0.354	0.731	51	0.408	0.155	0.611	52
Natural yogurt and quark	0.719	0.551	0.831	49	0.613	0.411	0.758	52
Flavored yogurt and quark	0.320	0.054	0.543	52	0.626	0.430	0.765	53
Puddings	0.576	0.356	0.735	50	0.423	0.175	0.621	53
Ice cream	0.149	-0.130	0.405	51	0.255	-0.014	0.490	53
**Cereal products**								
Sugar-sweetened cereals and muesli	0.550	0.333	0.711	54	0.533	0.311	0.699	54
Low- or non-sugar-containing cereals and muesli	0.503	0.267	0.683	51	0.461	0.220	0.649	53
Berry, fruit and chocolate porridge	0.195	-0.083	0.445	51	0.410	0.162	0.609	54
Sweet biscuits and cereal bars	0.577	0.362	0.733	52	0.620	0.423	0.762	53
Sweet pastries	0.324	0.059	0.547	52	0.419	0.173	0.616	54
**Drinks**								
Sugar-sweetened juice drinks	0.490	0.258	0.669	54	0.579	0.370	0.732	54
Soft drinks	0.567	0.350	0.726	52	0.499	0.269	0.675	54
Reduced sugar juices and soft drinks	0.000	-0.268	0.268	53	-0.009	-0.274	0.257	54
**Others**								
Chocolate	0.492	0.258	0.671	53	0.514	0.287	0.686	54
Sweets	0.384	0.132	0.590	54	0.349	0.092	0.562	54
Added sugar, honey or syrup	0.758	0.614	0.854	52	0.807	0.689	0.883	54
Jams, marmalades and sweetened spreads	0.516	0.280	0.694	50	0.582	0.374	0.734	54

* The guardians were asked to report how many times during the past week the child had consumed different foods at home or in places other than preschool.
